# Plasma proteomics-based liquid biopsy for predicting efficacy of PD-1-based immunochemotherapy in advanced gastric cancer: a prospective cohort study

**DOI:** 10.1186/s43556-026-00510-8

**Published:** 2026-07-07

**Authors:** Enqing Meng, Xu Cheng, Xinyi Wu, Linjun Wang, Xiaochun Ping, Mengxiao Wang, Minghui Ge, Xing Zhang, Dongsheng Chen, Chan Zhu, Ping Li, Hao Wu

**Affiliations:** 1 Department of Oncology, The First Affiliated Hospital of Nanjing Medical University, Nanjing, China; 2 Gastric Cancer Center, The First Affiliated Hospital of Nanjing Medical University, Nanjing, China; 3 Department of General Surgery, The First Affiliated Hospital of Nanjing Medical University, Nanjing, China; 4 State Key Laboratory of Neurology and Oncology Drug Development, Jiangsu Simcere Diagnostics Co., Ltd., Nanjing Simcere Medical Laboratory Science Co., Ltd., NanjingNanjing, Jiangsu China

**Keywords:** Gastric cancer, First-line immunochemotherapy, Liquid proteomic biopsy, Dynamic monitoring

## Abstract

The combination of immune checkpoint inhibitors (ICIs) and chemotherapy has become the standard first-line treatment for advanced gastric cancer and gastroesophageal junction cancer (GC/GEJC), yet reliable biomarkers predicting efficacy remain limited. We performed longitudinal plasma proteomic profiling in 31 patients receiving immunochemotherapy in this prospective observational cohort study. Serial plasma samples collected at baseline and every two treatment cycles were analyzed to identify dynamic protein changes correlated with therapeutic response and survival. The results showed that responders exhibited significantly lower baseline interleukin-15 (IL-15) levels (*P* = 0.01). After two cycles of treatment, a 161.9% reduction in plasma Mucin-16 (MUC-16), also known as Cancer Antigen-125 (CA-125), was identified as an independent factor for prolonged overall survival (HR 7.40, 95%CI 2.22–24.60, *P* = 0.001), which was validated by conventional serum CA-125 testing. Integrating baseline IL-15 with dynamic changes in MUC-16 and Matrix Metalloproteinase 12 (MMP12), the constructed composite risk model achieved an Area Under the Curve (AUC) of 0.799 for predicting response, and this model was also an independent factor for progression-free survival (HR 3.35, 95% 1.56–7.20, *P* = 0.002). In summary, baseline IL-15 helps identify patients likely to benefit from immunochemotherapy, while MUC-16 and the integrated model facilitate dynamic monitoring of therapeutic efficacy. These findings provide new insights into non-invasive blood-based patient stratification and treatment optimization.

**Trial registration** ChiCTR2300078000. Registered 27 November 2023—prospectively registered, https://www.chictr.org.cn/hvshowproject.html?id=241839&v=1.0

**Supplementary Information:**

The online version contains supplementary material available at 10.1186/s43556-026-00510-8.

## Introduction

Gastric cancer (GC) remains a leading cause of cancer mortality worldwide, with China accounting for over 40% of global cases [1, 2]. Although immune checkpoint inhibitors (ICIs) plus chemotherapy have been established as a cornerstone of first-line treatment for GC and gastroesophageal junction cancer (GEJC), and demonstrated significant survival benefits in randomized trials, clinical responses remain highly heterogeneous, with durable benefit achieved in only a subset of patients [3, 4]. Consequently, robust biomarkers for predicting therapeutic efficacy and survival outcomes are still needed.

Current tissue-based biomarkers, including microsatellite instability (MSI) [5], Epstein-Barr virus (EBV) [6], tumor mutational burden (TMB) [7], and PD-L1 expression identified only minority responders and are limited by tissue accessibility, intratumoral and temporal heterogeneity, and their largely static nature. Similarly, circulating tumor DNA (ctDNA) provides valuable information regarding tumor burden and clonal evolution but primarily reflects tumor-derived genomic alterations without capturing systemic immune status or treatment-induced immune modulation [8–10].

Blood biopsy approaches provide a minimally invasive complementary strategy [11, 12]. Circulating immune-related proteins integrate signals from tumor cells, immune cells, and the systemic inflammatory milieu, thereby capturing dynamic host-tumor immune interactions during immunotherapy. Longitudinal plasma proteomic enables monitoring treatment response and early resistance, with dynamic changes offering predictive value beyond static baseline markers [13, 14]. In gastrointestinal malignancies, circulating cytokines and tumor-associated proteins demonstrate prognostic relevance [15, 16], while plasma proteomics provides superior analytical stability compared to circulating tumor cells or ctDNA [17–19].

In this prospective, single-center study, we longitudinally profiled plasma immune-related proteins in patients with advanced GC/GEJC undergoing first-line PD-1 inhibitor based chemotherapy. We hypothesized that baseline circulating immune markers and early on-treatment protein dynamics reflect systemic immune responses to immunochemotherapy and are associated with treatment response and survival outcomes. By integrating baseline and dynamic plasma proteomic biomarkers, this study aims to identify clinically relevant, non-invasive predictors of immunochemotherapy efficacy and to provide insights into the systemic immune states underlying therapeutic response in advanced GC/GEJC.

## Results

### Clinical characteristics and plasma sample collection of patients

**Fig. 1:**
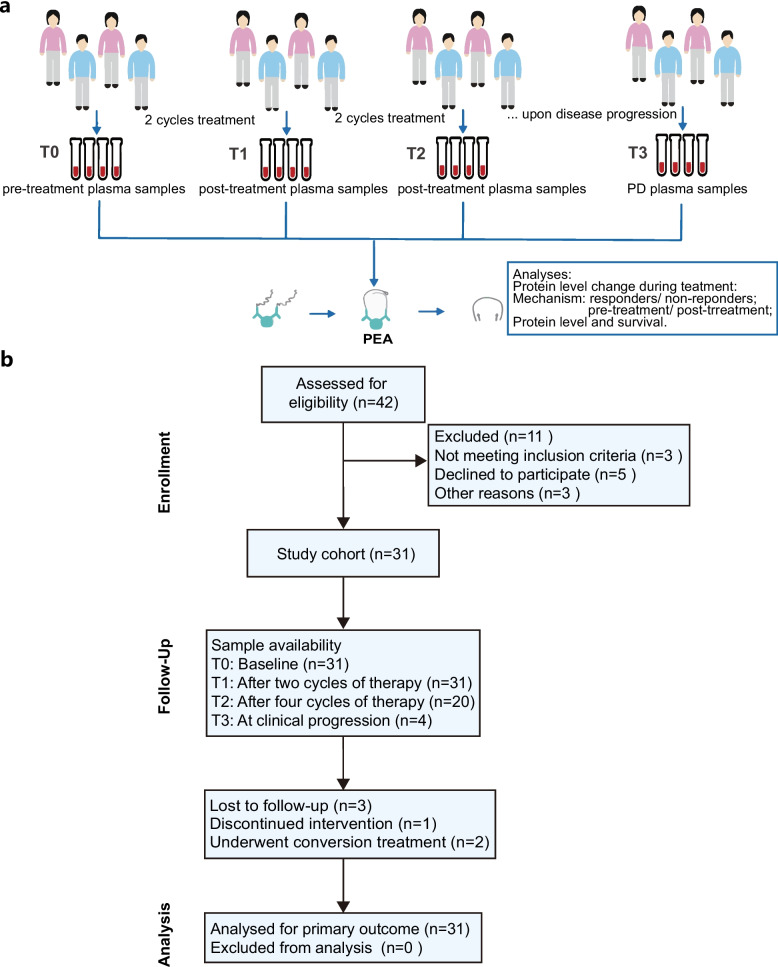
**a** Study workflow. In the study cohort, plasma samples were collected prior to treatment at baseline, at the end of every two cycles during treatment with ICIs combined with chemotherapy, and at PD. Treatment outcomes were followed prospectively. ICIs, immune checkpoint inhibitors; PD, progressive disease. **b** STROBE diagram for the study. STROBE, strengthening the reporting of observational studies in epidemiology

Forty-two patients were screened for eligibility, and a total of 31 eligible patients (median age, 67 years; range, 60.5–72.5 years) who received immunochemotherapy between May 2023 and September 2024 at the Oncology Department of the First Affiliated Hospital of Nanjing Medical University were enrolled. 31 plasma samples were collected at baseline and at T1, respectively. Plasma samples from 20 patients at T2 were collected, and 4 plasma samples were collected at T3 (Fig. 1a). STROBE diagram was presented in Fig. 1b. Patient baseline characteristics are detailed in Table 1, with 18 (58%) patients identified as responders (complete response plus primary response) and 13 (42%) as nonresponders (progressive disease plus stable disease) to the treatment. In this study, cohort, GC patients accounted for as many as 90.3% (28/31) of the cohort, and patients with gastroesophageal junction cancer accounted for 9.7% (3/31) of the cohort. In terms of the degree of histopathological differentiation, 12.9% (4/31) were moderately differentiated, and 45.2% (14/31) were poorly differentiated. Among all the patients, most advanced GC patients were diagnosed because of distant metastasis, with 77.4% (24/31), and patients with in situ recurrence after GC surgery accounted for 22.6% (7/31). Among the GC patients with distant metastasis, 45.2% (14/31) had liver metastasis, and 22.6% (7/31) had peritoneal metastasis.

**Table 1:** Characteristics of the study population

Characteristic	Overall	NR (non-responder)	R (responder)	*P*-value
n	31	13	18	
Age(years) (median (IQR))	67.00 (60.50,72.50)	67.00 (59.00,72.00)	67.00 (61.75,73.50)	0.810
Gender, n (%)				
Male	19 (61.3)	9 (69.2)	10 (55.6)	0.691
Female	12 (38.7)	4 (30.8)	8 (44.4)	
BMI (mean (SD))	21.68 (3.99)	21.54 (4.59)	21.78 (3.64)	0.873
ECOG performance status, n (%)				
0	13 (41.9)	5 (38.5)	8 (44.4)	1.000
1	18 (58.1)	8 (61.5)	10 (55.6)	
Cigarette smoking, n (%)	13 (41.9)	7 (53.8)	6 (33.3)	0.439
Alcohol consumption, n (%)	11 (35.5)	6 (46.2)	5 (27.8)	0.500
Family history of cancer, n (%)	5 (16.1)	3 (23.1)	2 (11.1)	0.690
Initial tumor diagnosis type, n (%)				
Gastric	28 (90.3)	10 (76.9)	18 (100.0)	0.126
Gastro-esophageal junction	3 (9.7)	3 (23.1)	0 (0.0)	
Differentiation type, n (%)				
Moderately differentiated	4 (12.9)	2 (15.4)	2 (11.1)	0.975
Moderately and poorly differentiated	3 (9.7)	1 (7.7)	2 (11.1)	
Poorly differentiated	14 (45.2)	6 (46.2)	8 (44.4)	
NA	10 (32.3)	4 (30.8)	6 (33.3)	
Lauren classification, n (%)				
Mixed	2 (6.5)	0 (0.0)	2 (11.1)	0.653
Diffuse	2 (6.5)	1 (7.7)	1 (5.6)	
Intestinal	4 (12.9)	2 (15.4)	2 (11.1)	
NA	23 (74.2)	10 (76.9)	13 (72.2)	
MSI status, n (%)	23 (74.2)	10 (76.9)	13 (72.2)	
MSS	19 (61.3)	7 (53.8)	12 (66.7)	0.727
MSI-High	0 (0.0)	0 (0.0)	0 (0.0)	
NA	12 (38.7)	6 (46.2)	6 (33.3)	
PD L1 expression, n (%)				
Positive	3 (9.7)	1 (7.7)	2 (11.1)	0.643
Negative	16 (51.6)	8 (61.5)	8 (44.4)	
NA	12 (38.7)	4 (30.8)	8 (44.4)	
Disease status, n (%)				
Locally recurrent	7 (22.6)	3 (23.1)	4 (22.2)	1.000
Metastatic	24 (77.4)	10 (76.9)	14 (77.8)	
Metastatic site, n (%)				
Liver	14 (45.2)	8 (61.5)	6 (33.3)	0.233
Lymph node	19 (61.3)	8 (61.5)	11 (61.1)	1.000
Peritoneum	7 (22.6)	3 (23.1)	4 (22.2)	1.000
Other	7 (22.6)	5 (38.5)	2 (11.1)	0.173
Number of metastatic sites, n (%)				
≤ 1	18 (58.0)	5 (38.5)	13 (72.2)	0.052
≥ 2	13 (42.0)	8 (61.5)	5 (27.8)	
Chemotherapy regimen, n (%)				
SOX	26 (83.9)	10 (76.9)	16 (88.9)	0.690
XELOX	5 (16.1)	3 (23.1)	2 (11.1)	

### Immunochemotherapy demonstrated variable therapeutic responses among patients

**Fig. 2:**
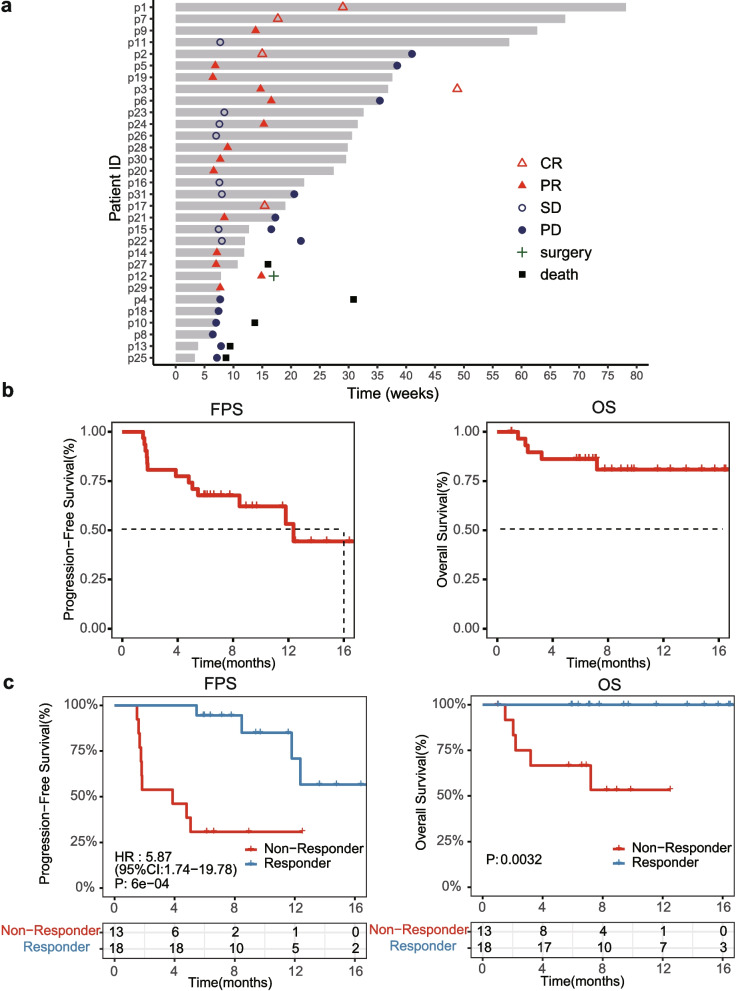
Clinical outcome results. **a** Inter heterogeneity in treatment response, disease course, and clinical outcomes. The length of each bar reflects follow-up time or time to progression (weeks). CR, complete response; PR, partial response; SD, stable disease; PD, progressive disease. Triangles, CR; red triangles, PR; circles, SD; crosses, surgical intervention; and black squares, death events. **b** Kaplan‒Meier survival curves for PFS and OS of all petients. **c** Kaplan‒Meier survival curves comparing PFS and OS between responders and non-responders. PFS, progression-free survival; OS, overall survival

The treatment response and clinical outcomes of 31 patients during treatment with this regimen are shown in Fig. 2a. Each patient is represented by a horizontal line, with key clinical events labeled along the timeline. Five (16.1%) patients achieved complete response (CR), with response times varying between approximately 10 and 40 weeks. Partial response (PR), which occurred more frequently, was observed in 13 (41.9%) patients, and all the cases appeared within the first 20 weeks of treatment. Stable disease (SD) was achieved in 7 (22.6%) patients. However, progressive disease (PD) eventually developed in 6 patients (19.4%), indicating that the patients became resistant to the therapy and that the disease progressed. In addition, 1 (3.2%) patient underwent surgery during the course of treatment when the tumor reached indications for resectability (Fig. 2a). Overall, the treatment response showed heterogeneity, with some patients achieving durable disease control, while others experienced early progression. These findings underscore the variable nature of the tumor response and the importance of personalized treatment strategies.

By September 23, 2024, the overall objective response rate (ORR) was 58.0% (95% CI, 39.1%–75.5%), the median PFS (mPFS) was 9.6 months (95% CI, 8.3-NA) (Fig. 2b), and the median OS (mOS) remained unreached during follow-up (Fig. 2b). The survival outcomes of responders and non-responders were compared. The mPFS for responders did not reach a certain value, while for non-responders, it was 3.87 months (HR 5.87, 95% CI: 1.74—19.78, *P* < 0.001); both the mOS for responders and non-responders did not reach (Fig. 2c). At the same time, the clinical characteristic analysis revealed that there was no bias between the responders and the non-responders (Table S1).

### Lower baseline Interleukin-15 (IL-15) level is associated with favorable immunochemotherapy response

**Fig. 3:**
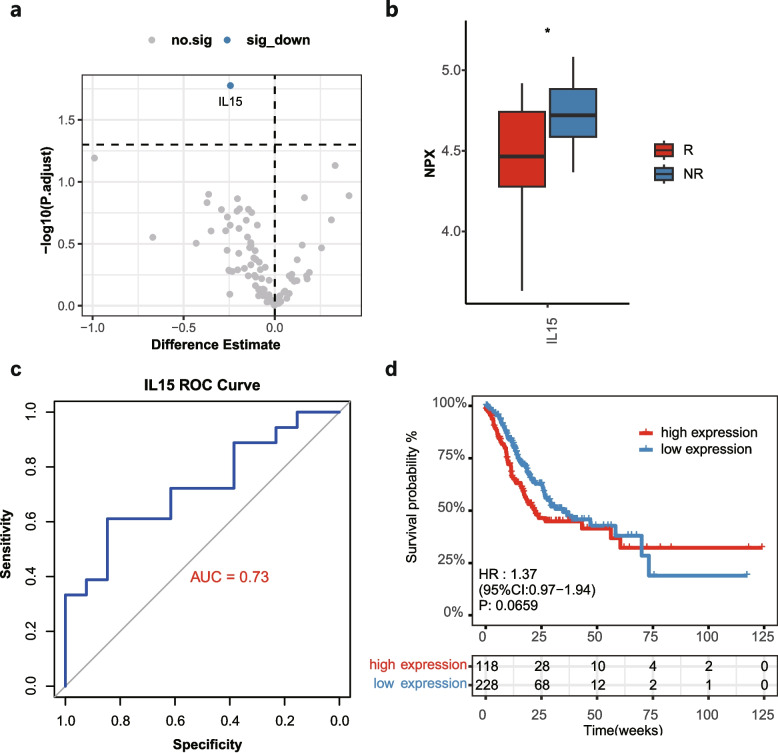
Baseline interleukin-15 (IL-15) levels correlate with treatment response and may predict resistance to immunochemotherapy. **a** and **b** Differences in baseline plasma protein levels between responders and non-responders. * *P* < 0.05. **c** ROC curve shows that pretreatment IL-15 expression was a good predictor of patient response to treatment. ROC, receiver operator characteristic curve. **d** Kaplan‒Meier survival curves demonstrating correlation between IL-15 expression level and patient survival in TCGA-STAD cohort. High vs. Low: 3.95 m vs. 8.7 m, HR 1.37, 95%CI: 0.97–1.94, *P* = *0.0659*. The Cancer Genome Atlas—Stomach Adenocarcinoma (TCGA-STAD)

The study comprised a total of 31 patients, responders (*N* = 18) exhibited significantly lower baseline plasma IL-15 levels compared with non-responders (*N* = 13) (Fig. 3a,b). An analysis was subsequently conducted to ascertain the accuracy of baseline IL-15 in predicting treatment response via the ROC model. The findings revealed a predicted AUC (Area Under the Curve) of 0.73 (Fig. 3c). Patients with low baseline IL-15 expression levels may be suitable candidates for this treatment, whereas those with high IL-15 expression levels may require alternative therapeutic interventions. The baseline IL-15 distribution didn’t exhibit population heterogeneity (Fig. S1). In The cancer genome atlas-Stomach adenocarcinoma (TCGA-STAD), IL-15 mRNA expression was not associated with OS and it suggested plasma IL-15 level may be the predicted biomarker for immunochemotherapy (Fig. 3d).

### Reduced post-treatment Mucin-16 (MUC-16) expression predicts better survival

**Fig. 4:**
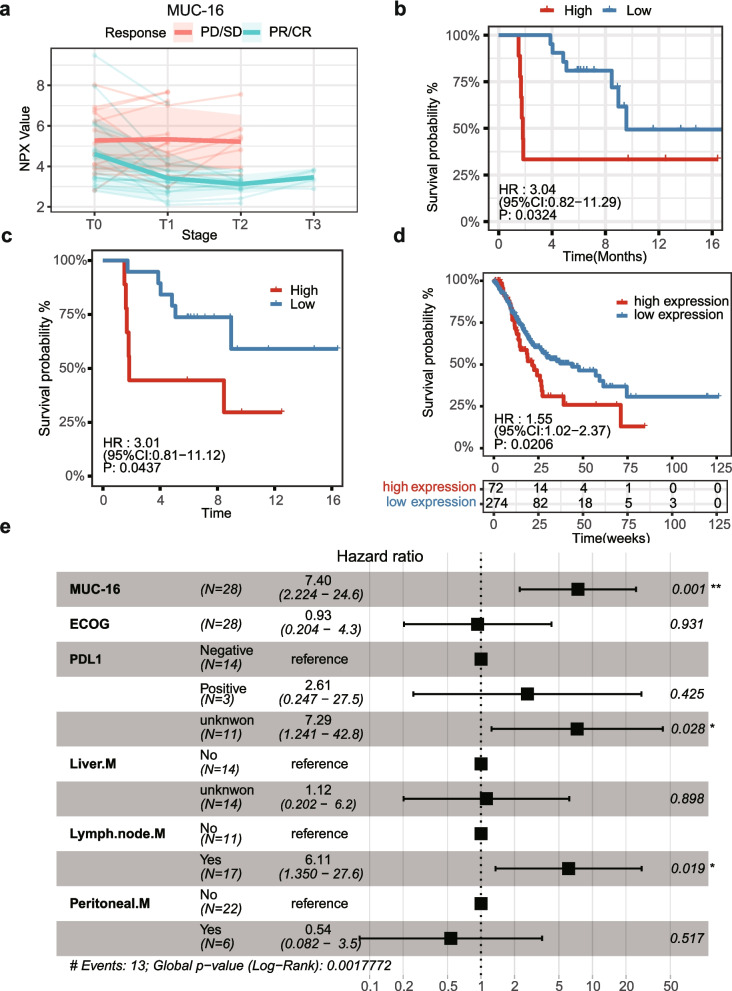
Post-treatment mucin-16 (MUC-16) dynamics predict clinical outcome. **a** Changes in plasma MUC-16 across treatment stages (T0; T1; T2; T3), stratified by clinical response (PR/CR vs SD/PD). Lines represent individual patients, with group-level trends overlaid. (T0: CR(5), PR(13), SD(7), PD(6); T1: CR(5), PR(13), SD(7), PD(6); T2: CR(4), PR(9), SD(7); T3: CR(1), PR(3)). CR, complete response; PR, partial response; SD, stable disease; and PD, progressive disease. **b** Reduced MUC-16 expression after treatment was associated with a better survival prognosis. High vs. Low: 9.58 m vs. 1.8 m, HR 3.04, 95%CI: 0.82%–11.29%, *P* = *0.032*. (T0, PD/SD: (Mean ± SEM:5.28 ± 0.44); T0, PR/CR: (Mean ± SEM: 4.61 ± 0.42); (T1, PD/SD: (Mean ± SEM:5.34 ± 0.44); T1, PR/CR: (Mean ± SEM: 3.41 ± 0.27); (T2, PD/SD: (Mean ± SEM:5.22 ± 0.50); T2, PR/CR: (Mean ± SEM: 3.13 ± 0.12); T3, PR/CR: (Mean ± SEM: 3.46 ± 0.22). CR, complete response; PR, partial response; SD, stable disease; and PD, progressive disease. **c** Reduced Cancer Antigen 125 (CA-125) after treatment was associated with a better survival prognosis. High vs. Low: 1.83 m vs. NA, HR 3.01, 95%CI: 0.81%–11.12%, *P* = 0.044. **d** Kaplan Meier survival curves demonstrating correlation between MUC-16 expression level and patient survival in TCGA-STAD cohort. High vs. Low: 11.3 m vs. 4.1 m, HR 1.55, 95%CI: 1.02%–2.37%, *P* = *0.0206*. The Cancer Genome Atlas—Stomach Adenocarcinoma (TCGA-STAD). **e** Multivariable Cox regression analysis assessing the independent prognostic value of MUC-16 dynamics after adjustment for clinical covariates. * *P* < 0.05, ** *P* < 0.01

Kaplan–Meier analysis revealed markers, including Matrix Metalloproteinase 12 (MMP12), Monocyte Chemoattractant Protein-4 (MCP-4), MUC-16, Monocyte Chemoattractant Protein-3 (MCP-3), Interleukin-4 (IL-4), and Interleukin-6 (IL-6), at multiple time points, as well as their on-treatment changes, were significantly associated with survival outcomes (Fig. S2). An examination of longitudinal expression patterns showed that, by the end of the second treatment cycle, the levels of these proteins generally declined in the majority of patients (Fig. 4a), suggesting a global modulation of systemic inflammatory and immune-related signals during early treatment. Notably, patients who exhibited an upregulation of MUC-16 following treatment experienced significantly shorter PFS compared with those whose MUC-16 levels decreased or remained stable (mPFS: 9.58 m vs. 1.8 m, HR 3.04, 95%CI: 0.82–11.29, *P* = 0.032, Fig. 4b).

We identified that a reduction in MUC-16 expression exceeding 161.9% at the initial efficacy assessment was associated with significantly improved PFS. The changes in Cancer Antigen 125 (CA-125) levels during the T0-T1 period were used to verify our results, which indicated that the reduction in CA-125 expression after treatment was associated with better survival (mPFS:1.8 m vs. NA, HR 3.01, 95%CI: 0.81–11.12, *P* = *0.044,* Fig. 4c). An independent survival analysis conducted in the TCGA-STAD cohort demonstrated that patients with lower tumor-associated MUC-16 expression had significantly prolonged OS (Fig. 4d). Importantly, this association remained statistically significant even after adjustment for established clinical covariates, underscoring the robustness of MUC-16 as an independent OS factor (HR 7.40, 95%CI 2.22–24.60, *P* = 0.001, Fig. 4e).

To address potential confounding from inflammation or infection, we assessed correlations between immunotherapy-associated plasma proteins and routine inflammatory indicators, including White Blood Cell (WBC), Neutrophil (NE), and Procalcitonin (PCT). While selected protein-marker pairs (e.g., MUC-16 with CA-125 and MMP12 with PCT) showed moderate to strong correlations at baseline and during treatment, these associations were protein-specific rather than global, arguing against a nonspecific inflammation-driven effect. Notably, serum CA-125 (also known as MUC-16) levels revealed significantly correlated with plasma MUC-16 expression (R = 0.905, *P* < 0.001), further supports the reliability of plasma proteomic measurements (Fig. S3).

### A longitudinal plasma proteomic model predicts therapeutic efficacy

The present study seeks to explore the potential guiding role of peripheral protein changes in the clinical treatment of patients with advanced GC, we established a joint risk model with the efficacy prediction markers and prognostic related markers screened in the previous stage. And performed functional enrichment of the protein changed before and after treatment.

**Fig. 5:**
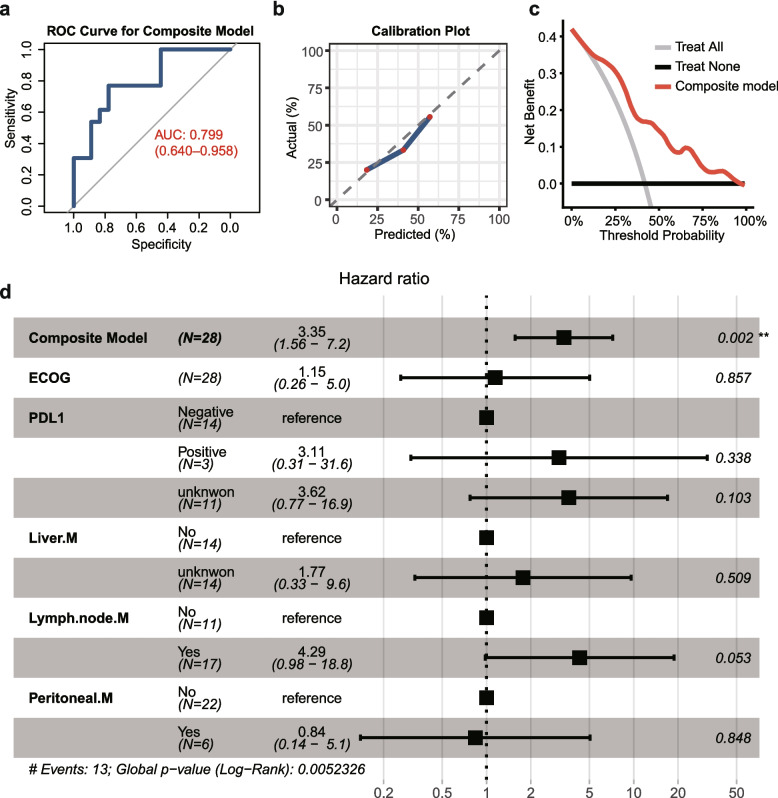
Plasma protein alterations inform clinical decision-making. **a** ROC curve of a LASSO model combining baseline interleukin-15 (IL-15) and changes in matrix metalloproteinase 12 (MMP12) and mucin-16 (MUC-16). Predictive performance and survival relevance of the composite plasma proteomic model. ROC, receiver operator characteristic curve. ROC, receiver operator characteristic curve; LASSO, least absolute shrinkage and selection operator. **b** Calibration plot comparing predicted probabilities with observed response rates. **c** DCA illustrating the net clinical benefit of the composite model across a range of threshold probabilities. DCA, decision curve analysis. **d** Multivariable Cox proportional hazards analysis evaluating the association between the composite risk score and progression-free survival. Hazard ratios with 95% confidence intervals are shown. ** *P* < 0.01

A predictive signature was constructed using Least Absolute Shrinkage and Selection Operator (LASSO) logistic regression based on three optimized features: baseline IL-15, and the dynamic changes (Δ) of MUC-16 and MMP12. These three markers showed no subgroup differences within the cohort at T0 (Table S2). The final LASSO model demonstrated a strong apparent discriminatory power with an apparent AUC of 0.865 (95%CI: 0.724–1.000), yielding a cross-validated AUC of 0.799 (95%CI: 0.640–0.958) (Fig. 5a). The optimal tuning parameter ℷ was identified as 0.044 via tenfold cross-validation. The risk score was derived as follows:

$$\begin{aligned}&\mathrm{Risk\;Score}=-5.948+(1.287\times\mathrm{IL}{15}\_{\mathrm{baseline}})\\&+(0.021\times\Delta\mathrm{MUC}-16)+(0.064\times\Delta\mathrm{MMP}12)\end{aligned}$$

Calibration analysis indicated close agreement between predicted and observed risks (Fig. 5b). Decision curve analysis showed greater net benefit of the composite model across clinically relevant threshold probabilities (approximately 10%–70%) (Fig. 5c). Importantly, it remained an independent predictor of outcome in multivariable Cox analysis after adjustment for established clinical factors, supporting its potential utility for risk stratification beyond conventional markers (Fig. 5d). These findings indicate that the combined plasma protein signature may serve as a useful predictor of treatment response.

All proteins quantified in the Olink Target 96 IO panel were performed GO functional annotation analysis between responders and non-responders. Several different expression genes, including IL15, MMP12, and Vascular Endothelial Growth Factor A (VEGFA)-the data demonstrated a substantial enrichment in biological processes (GO-BP), including neutrophil migration, granulocyte chemotaxis, and humoral immune response, processes known to facilitate vascular remodeling and immune cell recruitment [20–22]. To translate these findings into potential clinical applications, we next constructed a drug-gene interaction network (Fig. S4). IL15 and MMP12 were associated with immune-modulatory agents such as sirolimus, adalimumab, and thalidomide, suggesting that these molecules may represent key immunovascular signaling nodes suitable for combination targeting.

## Discussion

In this study, we evaluated the efficacy and potential biomarkers of first-line immunochemotherapy in patients with advanced GC and explored plasma protein biomarkers that may aid in response prediction and treatment stratification. Using plasma proteomics, we identified three promising biomarkers, including IL-15, MUC-16, and MMP12, that may support treatment stratification and prognosis prediction.

Among these markers, responders exhibited significantly lower baseline IL-15 levels than non-responders, suggesting its utility as a predictive marker of immunochemotherapy efficacy. IL-15 promotes CD8 + T-cell and natural killer cell survival and activation. Recent studies demonstrate that IL-15 signaling within tumor cells can promote pro-tumorigenic behaviors, even while exogenous IL-15 administration may sensitize tumors to checkpoint inhibition, highlighting the complexity of IL-15’ s impact in vivo [23–25]. Accumulating evidence suggests that sustained cytokine exposure can contribute to immune dysregulation, including T-cell exhaustion and impaired effector function, which may limit responsiveness to immune checkpoint blockade [26, 27]. Patients with low IL-15 expression may benefit more from combination therapy due to restored immune responsiveness. These results suggest that IL-15 may serve as a non-invasive biomarker to stratify patients before initiating immunochemotherapy. In light of the exploratory design, the potential predictive value of IL-15 should be interpreted cautiously and requires validation in larger cohorts.

MUC-16, also known as CA-125, is a transmembrane glycoprotein that is overexpressed in a variety of tumors, including GC [28]. Serum CA-125 levels have been generally used for the diagnosis and effective monitoring of ovarian cancer and lung cancer [29, 30]. Our study demonstrated that plasma MUC-16, declined significantly in patients with longer PFS, suggesting that early downregulation may indicate favorable prognosis. Moreover, some studies have reported that high MUC-16 expression pancreatic cancer patients have a worse prognosis [31, 32], supporting our findings that its downregulation after treatment is associated with a better prognosis. These findings suggest that patients with advanced GC with reduced plasma MUC-16 have a better prognosis in first-line immunochemotherapy [33].

The model incorporating IL-15, MMP12 and MUC-16 is promising to predict the response and prognosis in advanced GC patients undergoing immunochemotherapy. Tissue-specific biomarkers like PD-L1 and MSI represent static tumor features [34, 35]. While relative to existing blood-based markers, deep sequencing in longitudinal monitoring is employed to profile a broader array of tumor-immune-related proteins in our study, and partial discovery is validated by CA-125. Pretreatment IL-15 level could contribute to identify patients likely to benefit, MUC-16 kinetics enable dynamic monitoring of therapeutic efficacy, and a model incorporating IL-15, MUC-16, and MMP12 has the potential to predict early response. Importantly, the feasibility, repeatability, and rapid turnaround of plasma-based assays make them well suited for real-world implementation. Due to the significantly strong correlations between the MUC-16 expression by plasma and serum CA-125 level in our study, it suggests that biomarkers identified through high-throughput screening have the potential to be translated into absolutely quantitative and clinically routine assays. While our findings provide preliminary clinical evidence linking these biomarkers to outcomes, mechanistic studies and experimental validation are warranted to clarify their biological roles and to strengthen their translational relevance.

Despite encouraging results, several limitations of this study should be acknowledged. Our findings should be interpreted as exploratory and hypothesis-generating, therefore do not imply a direct causal relationship between the identified biomarkers and treatment response or clinical outcomes. First, the relatively modest sample size may curtail statistical power. Second, although internal validation using cross-validation and bootstrapping was performed, the lack of an independent external cohort prevents definitive assessment of model robustness and clinical reproducibility. We plan to validate the identified plasma biomarkers in larger, independent multicenter cohorts with systematic sample collection and standardized analytical pipelines. Prospective validation across diverse patient populations and clinical settings to confirming clinical utility and generalizability. We also planned to establish new decision-making criteria by integrating the existing clinical markers (eg. PD-L1, MSI, and ctDNA). Third, TCGA analysis can only provide indirect supplementation for the plasma proteomics findings in this study due to the heterogeneity between tissues. For the TCGA survival analysis did not undergo multiple validations, the results are exploratory. Finally, standardized absolute quantification protocols and cost-effective detection platforms are still needed to translate these biomarkers and models. Further studies are warranted to validate causality and clinical value requires confirmation in multi-center cohorts with larger samples.

In conclusion, dynamic circulating immune-related proteins can capture systemic immunological changes during immunochemotherapy in GC/GEJC. Our study reveals the clinical value of baseline IL-15 in predicting treatment response, MUC-16 (CA-125) kinetics, and a model incorporating IL-15, MUC-16, and MMP12 in efficacy dynamic monitoring. It suggests that minimally invasive, repeatable plasma biomarkers have the potential to inform real-time treatment stratification and optimize immunochemotherapy decision-making.

## Material and method

### Study design

This prospective cohort study was conducted at the Oncology Department of the First Affiliated Hospital of Nanjing Medical University between 2023 and 2024. Institutional review board approval was obtained before study initiation, and all procedures complied with the Declaration of Helsinki and local regulatory standards. The inclusion and exclusion criteria of patients, regimen procedures and outcome estimation were shown in Supplementary Material 1. All patients provided written informed consent.

### Patients

Eligible patients were aged 18 years or older with histologically confirmed, unresectable, locally advanced, recurrent, or metastatic GC/GEJ. Patients who relapsed following neoadjuvant or adjuvant therapy were eligible provided there was a treatment-free interval of more than 6 months between the completion of therapy and recurrence. Eligibility required an Eastern Cooperative Oncology Group (ECOG) performance status of 0–1 and ≥ 1 measurable lesion per the Response Evaluation Criteria in Solid Tumors Version 1.1 (RECIST 1.1). Patients were excluded if they had other active malignancies within 5 years or currently, except for completely cured localized malignancies. Patients with a known history of severe hypersensitivity to monoclonal antibodies or any study drug components were excluded.

### Sample collection and follow-up

Plasma samples (approximately 5 ml of blood) were collected at baseline (T0), at the end of cycle 2 (T1), at the end of cycle 4 (T2) and in the event of disease progression (T3). Samples were collected from 31 patients at baseline and at T1, respectively. Due to PD, death, or loss to follow-up, samples from 20 patients at T2 were collected. Analyses involving cycle-4 measurements were restricted to patients with available data. The number of patients included in each analysis is indicated in the corresponding figure legends. For dynamic biomarkers, a landmark analysis was conducted at T1. Only patients available at T1 (*n* = 25) were included in subsequent survival analyses of dynamic change metrics. Similar landmark definitions were applied at T2.

If the patient's condition was stable, after completing 6 courses of treatment, follow-up was performed every 3 months, as follows: (1) routine examination: medical history, physical examination, blood tests, and imaging; (2) blood collection and protein testing were completed within one week when symptoms worsened or new symptoms appeared.

### Plasma differential expression protein assay

Plasma protein profiling used Olink Target 96 Immuno-Oncology (IO) panel (Olink Proteomics, Uppsala, Sweden), which enables multiplexed, antibody-based proximity extension assay (PEA) technology to quantify 92 immune-related proteins simultaneously (Table S3). Briefly, plasma samples were incubated with oligonucleotide-labeled antibodies; binding enabled DNA polymerase extension, generating protein-specific PCR amplicons. Quantification was performed by real-time PCR; three internal controls were spiked per sample, with external controls included on each plate for quality assurance. Ct values were quality controlled and normalized using three negative controls to estimate LOD and three plate controls (PCs) containing 92 antibodies; assay results were reported as log2-transformed normalized protein expression (NPX) values calculated using the specified formula: target dCt = target Ct-negative control Ct-interplate control Ct; target ddCt = correction factor (reagent batch variable)—target dCt.

Data were normalized using Olink’s standard normalization procedure. NPX values were log2-transformed prior to downstream statistical analyses. Proteins failing quality control thresholds were excluded (standard deviation of internal controls < 0.2 NPX). Given the approximately symmetric distribution of NPX values and prior evidence supporting the robustness of the Student’s t-test in log-transformed omics data, parametric tests were used for exploratory comparisons between groups. The complete list of analyzed proteins is provided in Table S3. The TCGA data acquisition and analysis and predictive model construction were shown in Supplementary Material 1.

### Statistic analysis

This study was designed as a prospective exploratory analysis. Given its hypothesis-generating nature, formal power calculations were not performed. According to the suggested sample size of 20–50 in exploratory studies [11], we included 31 patients in our trial. Dynamic changes in plasma protein levels were defined as the relative percentage change from baseline at each subsequent sampling time point: (T1 − T0)/T0 × 100%. For response-associated analyses, optimal cut-off values for dynamic changes were determined through ergodic analysis, with better survival as the classification outcome.

Primary characteristics were summarized as frequencies/proportions or medians (IQRs, ranges); normality was assessed using the Shapiro–Wilk test. For unpaired comparisons, Student’s t-test was used for normally distributed data and Wilcoxon rank-sum test for non-normal data. Categorical variables were summarized as frequencies and percentages and compared using λ^2^ test or Fisher’s exact test. *P* values less than 0.05 were considered to be statistically significant. Difference estimate was calculated as log2 fold change of NPX between 2 groups. Olink NPX Signature 1.5.3.0 software was used to analyze the data, and the criterion for determining differentially expressed proteins was *P* < 0.05 for the comparison of NPX between the two groups, with a difference of > 0 defined as upregulation and < 0 defined as downregulation. Expression profiles of 96 plasma proteins with survival data were analyzed using Cox regression and Kaplan–Meier methods. Analyses were performed using GraphPad 9.0, R 4.1, and R Bioconductor packages (https://www.r-project.org).

## Supplementary Information

Supplementary Material 1.

## Data Availability

All data were included in this paper and deposited online: 10.5281/zenodo.18297346.

## References

[1] Bray F, Laversanne M, Sung H, Ferlay J, Siegel RL, Soerjomataram I, et al. Global cancer statistics 2022: GLOBOCAN estimates of incidence and mortality worldwide for 36 cancers in 185 countries. CA Cancer J Clin. 2024;74(3):229–63. 10.3322/caac.21834. 38572751 10.3322/caac.21834

[2] Li Y, Feng A, Zheng S, Chen C, Lyu J. Recent estimates and predictions of 5-year survival in patients with gastric cancer: a model-based period analysis. Cancer Control. 2022;29:10732748221099227. 10.1177/10732748221099227. 35499497 10.1177/10732748221099227 PMC9067041

[3] Janjigian YY, Shitara K, Moehler M, Garrido M, Salman P, Shen L, et al. First-line nivolumab plus chemotherapy versus chemotherapy alone for advanced gastric, gastro-oesophageal junction, and oesophageal adenocarcinoma (CheckMate 649): a randomised, open-label, phase 3 trial. Lancet. 2021;398(10294):27–40. 10.1016/S0140-6736(21)00797-2. 34102137 10.1016/S0140-6736(21)00797-2 PMC8436782

[4] Xu J, Jiang H, Pan Y, Gu K, Cang S, Han L, et al. Sintilimab plus chemotherapy for unresectable gastric or gastroesophageal junction cancer: the ORIENT-16 randomized clinical trial. JAMA. 2023;330(21):2064–74. 10.1001/jama.2023.19918. 38051328 10.1001/jama.2023.19918 PMC10698618

[5] Marcus L, Lemery SJ, Keegan P, Pazdur R. FDA approval summary: pembrolizumab for the treatment of microsatellite instability-high solid tumors. Clin Cancer Res. 2019;25(13):3753–8. 10.1158/1078-0432.CCR-18-4070. 30787022 10.1158/1078-0432.CCR-18-4070

[6] Kim ST, Cristescu R, Bass AJ, Kim KM, Odegaard JI, Kim K, et al. Comprehensive molecular characterization of clinical responses to PD-1 inhibition in metastatic gastric cancer. Nat Med. 2018;24(9):1449–58. 10.1038/s41591-018-0101-z. 30013197 10.1038/s41591-018-0101-z

[7] Marabelle A, Fakih M, Lopez J, Shah M, Shapira-Frommer R, Nakagawa K, et al. Association of tumour mutational burden with outcomes in patients with advanced solid tumours treated with pembrolizumab: prospective biomarker analysis of the multicohort, open-label, phase 2 KEYNOTE-158 study. Lancet Oncol. 2020;21(10):1353–65. 10.1016/S1470-2045(20)30445-9. 32919526 10.1016/S1470-2045(20)30445-9

[8] Jin Y, Chen DL, Wang F, Yang CP, Chen XX, You JQ, et al. The predicting role of circulating tumor DNA landscape in gastric cancer patients treated with immune checkpoint inhibitors. Mol Cancer. 2020;19(1):154. 10.1186/s12943-020-01274-7. 33126883 10.1186/s12943-020-01274-7 PMC7596978

[9] Cabel L, Proudhon C, Romano E, Girard N, Lantz O, Stern MH, et al. Clinical potential of circulating tumour DNA in patients receiving anticancer immunotherapy. Nat Rev Clin Oncol. 2018;15(10):639–50. 10.1038/s41571-018-0074-3. 30050094 10.1038/s41571-018-0074-3

[10] Zulato E, Del Bianco P, Nardo G, Attili I, Pavan A, Boscolo Bragadin A, et al. Longitudinal liquid biopsy anticipates hyperprogression and early death in advanced non-small cell lung cancer patients treated with immune checkpoint inhibitors. Br J Cancer. 2022;127(11):2034–42. 10.1038/s41416-022-01978-1. 36175621 10.1038/s41416-022-01978-1 PMC9681746

[11] Kok VC, Yu CC. Cancer-Derived Exosomes: Their Role in Cancer Biology and Biomarker Development. Int J Nanomedicine. 2020;15:8019–36. 10.2147/IJN.S272378. 33116515 10.2147/IJN.S272378 PMC7585279

[12] Dasari A, Morris VK, Allegra CJ, Atreya C, Benson AB 3rd, Boland P, et al. ctDNA applications and integration in colorectal cancer: an NCI Colon and Rectal-Anal Task Forces whitepaper. Nat Rev Clin Oncol. 2020;17(12):757–70. 10.1038/s41571-020-0392-0. 32632268 10.1038/s41571-020-0392-0 PMC7790747

[13] Gao M, Wu X, Jiao X, Hu Y, Wang Y, Zhuo N, et al. Prognostic and predictive value of angiogenesis-associated serum proteins for immunotherapy in esophageal cancer. J Immunother Cancer. 2024;12(2):e006616. 10.1136/jitc-2022-006616. 38302415 10.1136/jitc-2022-006616 PMC10836376

[14] Gao Y, Qi F, Zhou W, Jiang P, Hu M, Wang Y, et al. Liquid biopsy using plasma proteomics in predicting efficacy and tolerance of PD-1/PD-L1 blockades in NSCLC: a prospective exploratory study. Mol Biomed. 2025;6(1):51. 10.1186/s43556-025-00291-6. 40659985 10.1186/s43556-025-00291-6 PMC12260144

[15] Sokolova O, Naumann M. Matrix metalloproteinases in *Helicobacter pylori*-associated gastritis and gastric cancer. Int J Mol Sci. 2022;23(3):1883. 10.3390/ijms23031883. 35163805 10.3390/ijms23031883 PMC8836485

[16] Nakanishi T, Imano M, Kohda M, Kato H, Kounami N, Yamada A, et al. Intraperitoneal immune microenvironment and efficacy of intraperitoneal chemotherapy in patients with gastric cancer and peritoneal metastasis. Sci Rep. 2025;15(1):44193. 10.1038/s41598-025-27936-4. 41419554 10.1038/s41598-025-27936-4 PMC12717035

[17] Cierna Z, Smolkova B, Cholujova D, Gronesova P, Miklikova S, Cihova M, et al. Decreased levels of circulating cytokines VEGF, TNF-beta and IL-15 indicate PD-L1 overexpression in tumours of primary breast cancer patients. Sci Rep. 2021;11(1):1294. 10.1038/s41598-020-80351-9. 33446741 10.1038/s41598-020-80351-9 PMC7809365

[18] Tan Q, Gao R, Zhang X, Yang J, Xing P, Yang S, et al. Longitudinal plasma proteomic analysis identifies biomarkers and combinational targets for anti-PD1-resistant cancer patients. Cancer Immunol Immunother. 2024;73(3):47. 10.1007/s00262-024-03631-7. 38349411 10.1007/s00262-024-03631-7 PMC10864508

[19] Sun J, Li X, Wang Q, Chen P, Zhao L, Gao Y. Proteomic profiling and biomarker discovery for predicting the response to PD-1 inhibitor immunotherapy in gastric cancer patients. Front Pharmacol. 2024;15:1349459. 10.3389/fphar.2024.1349459. 38881867 10.3389/fphar.2024.1349459 PMC11176556

[20] Bergamaschi C, Pandit H, Nagy BA, Stellas D, Jensen SM, Bear J, et al. Heterodimeric IL-15 delays tumor growth and promotes intratumoral CTL and dendritic cell accumulation by a cytokine network involving XCL1, IFN-gamma, CXCL9 and CXCL10. J Immunother Cancer. 2020;8(1):e000599. 10.1136/jitc-2020-000599. 32461349 10.1136/jitc-2020-000599 PMC7254133

[21] Seo IH, Eun HS, Kim JK, Lee H, Jeong S, Choi SJ, et al. IL-15 enhances CCR5-mediated migration of memory CD8(+) T cells by upregulating CCR5 expression in the absence of TCR stimulation. Cell Rep. 2021;36(4):109438. 10.1016/j.celrep.2021.109438. 34320338 10.1016/j.celrep.2021.109438

[22] Kerkela E, Ala-aho R, Klemi P, Grenman S, Shapiro SD, Kahari VM, et al. Metalloelastase (MMP-12) expression by tumour cells in squamous cell carcinoma of the vulva correlates with invasiveness, while that by macrophages predicts better outcome. J Pathol. 2002;198(2):258–69. 10.1002/path.1198. 12237887 10.1002/path.1198

[23] Fiore PF, Di Matteo S, Tumino N, Mariotti FR, Pietra G, Ottonello S, et al. Interleukin-15 and cancer: some solved and many unsolved questions. J Immunother Cancer. 2020;8(2):e001428. 10.1136/jitc-2020-001428. 33203664 10.1136/jitc-2020-001428 PMC7674108

[24] Barzegar C, Meazza R, Pereno R, Pottin-Clemenceau C, Scudeletti M, Brouty-Boye D, et al. IL-15 is produced by a subset of human melanomas, and is involved in the regulation of markers of melanoma progression through juxtacrine loops. Oncogene. 1998;16(19):2503–12. 10.1038/sj.onc.1201775. 9627116 10.1038/sj.onc.1201775

[25] Kuniyasu H, Ohmori H, Sasaki T, Sasahira T, Yoshida K, Kitadai Y, et al. Production of interleukin 15 by human colon cancer cells is associated with induction of mucosal hyperplasia, angiogenesis, and metastasis. Clin Cancer Res. 2003;9(13):4802–10. 10.1117/12.869070. 14581351

[26] Zhang R, Jiang Q, Guo R, Guo K, Qiu J. Unveiling the power of plumbagin: revitalizing exhausted T cells to combat tongue cancer. Cancer Cell Int. 2025;25(1):271. 10.1186/s12935-025-03892-x. 40684194 10.1186/s12935-025-03892-x PMC12276689

[27] Newman MJ. Invention and characterization of a systemically administered, attenuated and killed bacteria-based multiple immune receptor agonist for anti-tumor immunotherapy. Front Immunol. 2024;15:1462221. 10.3389/fimmu.2024.1462221. 39606250 10.3389/fimmu.2024.1462221 PMC11599860

[28] Lakshmanan I, Marimuthu S, Chaudhary S, Seshacharyulu P, Rachagani S, Muniyan S, et al. Muc16 depletion diminishes KRAS-induced tumorigenesis and metastasis by altering tumor microenvironment factors in pancreatic ductal adenocarcinoma. Oncogene. 2022;41(48):5147–59. 10.1038/s41388-022-02493-6. 36271032 10.1038/s41388-022-02493-6 PMC9841597

[29] Szymanska B, Lukaszewski Z, Hermanowicz-Szamatowicz K, Gorodkiewicz E. A biosensor for determination of the circulating biomarker CA125/MUC16 by Surface Plasmon Resonance Imaging. Talanta. 2020;206:120187. 10.1016/j.talanta.2019.120187. 31514860 10.1016/j.talanta.2019.120187

[30] Lung Cancer Cohort C. The blood proteome of imminent lung cancer diagnosis. Nat Commun. 2023;14(1):3042. 10.1038/s41467-023-37979-8. 37264016 10.1038/s41467-023-37979-8 PMC10235023

[31] Wang S, You L, Dai M, Zhao Y. Mucins in pancreatic cancer: a well-established but promising family for diagnosis, prognosis and therapy. J Cell Mol Med. 2020;24(18):10279–89. 10.1111/jcmm.15684. 32745356 10.1111/jcmm.15684 PMC7521221

[32] Liu C, Deng S, Jin K, Gong Y, Cheng H, Fan Z, et al. Lewis antigen‑negative pancreatic cancer: an aggressive subgroup. Int J Oncol. 2020;56(4):900–8. 10.3892/ijo.2020.4989. 32319567 10.3892/ijo.2020.4989 PMC7050983

[33] Zhang L, Han X, Shi Y. Association of MUC16 mutation with response to immune checkpoint inhibitors in solid tumors. JAMA Netw Open. 2020;3(8):e2013201. 10.1001/jamanetworkopen.2020.13201. 32845327 10.1001/jamanetworkopen.2020.13201 PMC7450349

[34] Hwang M, Canzoniero JV, Rosner S, Zhang G, White JR, Belcaid Z, et al. Peripheral blood immune cell dynamics reflect antitumor immune responses and predict clinical response to immunotherapy. J Immunother Cancer. 2022;10(6):e004688. 10.1136/jitc-2022-004688. 35688557 10.1136/jitc-2022-004688 PMC9189831

[35] Kaczmarek F, Marcinkowska-Gapinska A, Bartkowiak-Wieczorek J, Nowak M, Kmiecik M, Brzezinska K, et al. Blood-Based Biomarkers as Predictive and Prognostic Factors in Immunotherapy-Treated Patients with Solid Tumors-Currents and Perspectives. Cancers (Basel). 2025;17(12):2001. 10.3390/cancers17122001. 40563651 10.3390/cancers17122001 PMC12190272

