# Supplementary material for: Plasma proteomics-based liquid biopsy for predicting efficacy of PD-1-based immunochemotherapy in advanced gastric cancer: a prospective cohort study
Source: Mol Biomed. 2026 Jul 7;7:106. doi: 10.1186/s43556-026-00510-8 (PMC13341996; doi:10.1186/s43556-026-00510-8)
Supplement: Supplementary file 1 — Supplementary Material 1. [file 43556_2026_510_MOESM1_ESM.docx]

**Plasma proteomics-based liquid biopsy for predicting efficacy of PD-1-based immunochemotherapy in advanced gastric cancer: a prospective cohort study**

Enqing Meng^1,#^, Xu Cheng^1^, Xinyi Wu^1^, Linjun Wang^2,3^, Xiaochun Ping^2,3^, Mengxiao Wang^4^, Minghui Ge^4^, Xing Zhang^4^, Dongsheng Chen^4^, Chan Zhu^4^, PingLi^1,2,*^, Hao Wu^1,2,*^

^1^Department of Oncology, The First Affiliated Hospital of Nanjing Medical University, Nanjing, China

^2^Gastric Cancer Center, The First Affiliated Hospital of Nanjing Medical University, Nanjing, China

^3^Department of General Surgery, The First Affiliated Hospital of Nanjing Medical University, Nanjing, China

^4^State Key Laboratory of Neurology and Oncology Drug Development, Jiangsu Simcere Diagnostics Co., Ltd., Nanjing Simcere Medical Laboratory Science Co., Ltd., Nanjing, Jiangsu, China

Corresponding author:

**Hao Wu**: [whdactor@njmu.edu.cn](mailto:whdactor@njmu.edu.cn)

**Ping Li:** tzliping@163.com

# First author

* The corresponding author

**Methods**

**Ethics**

The present study was conducted in accordance with the Declaration of Helsinki. Written informed consent was obtained from all participants. The study was approved by the Ethics Committee of the First Affiliated Hospital of Nanjing Medical University (approval number 2023-SR-353). Informed written consent was obtained from each subject or independent witness prior to enrollment in the clinical trial. This study was registered with the China Clinical Trial Registry (ChiCTR2300078000).

**Procedures**

The participants received a combination therapy of sintilimab and chemotherapy, with the chemotherapy regimen including two options: the SOX regimen (oxaliplatin and S-1) and the XELOX regimen (oxaliplatin and capecitabine). A total of 6 cycles of the combined therapy were administered, with each cycle lasting 21 days. After completing 6 cycles of combination therapy, the participants proceeded to maintenance therapy with sintilimab plus an oral chemotherapy agent, either capecitabine or S-1, which continued until disease progression.

Imaging assessments (computed tomography (CT)/ magnetic resonance imaging (MRI)) were required prior to the initial treatment and every two cycles of treatment.

**Outcomes**

Tumour response was assessed by radiologists who were blinded to proteomic data through radiological evaluation (CT or MRI) according to the Response Evaluation Criteria in Solid Tumors (RECIST) v1.1. Assessments were conducted at baseline and every 6 weeks (two cycles) thereafter. Patients were categorized as responders if they achieved a best overall response of complete response or partial response, typically documented within two months of treatment initiation; non-responders were defined as those with stable disease or progressive disease. The clinical endpoints were overall response rate (defined as the proportion of patients who achieved a complete or partial response), progression-free survival (defined as the time from the start of treatment to the first documented objective disease progression per RECIST 1.1 or death from any cause), and overall survival (defined as the time from the start of treatment to death from any cause).

**TCGA data acquisition and analysis**

Transcriptomic expression data for TCGA-STAD were retrieved from The Human Protein Atlas (HPA) (https://www.proteinatlas.org). For survival analysis, HPA leverages the TCGA-STAD cohort. The mRNA expression levels were quantified as Fragments Per Kilobase of exon per Million reads (FPKM). The optimal expression cut-off for dichotomizing patients into "High" and "Low" expression groups was determined using the "Best Expression Cut-off" method. This approach involves a systematic scan of all potential FPKM values to identify the threshold that yields the minimal p-value in a Log-rank test, thereby maximizing the statistical significance of survival differences between the two subgroups. The optimal expression cut-off was determined using the minimum p-value approach, which evaluates all possible thresholds to identify the value that best separates survival outcomes. No multiple testing correction was applied during this process; therefore, the results should be interpreted with caution. This cut-off was derived in an exploratory manner and has not been externally validated.

**Predictive Model Construction and Validation**

To develop a robust predictive signature, we employed a Least Absolute Shrinkage and Selection Operator (LASSO) logistic regression using the glmnet R package. The baseline IL-15 levels and the percentage changes（Δ）of MUC-16 and MMP12 were utilized as input features. To determine the optimal tuning parameter（ℷ）, we performed 10-fold cross-validation using the cv.glmnet function, selecting the ℷ that maximized the Area Under the Receiver Operating Characteristic curve (AUC)

To mitigate the risk of overfitting given the limited sample size (n=31), the model's performance was further evaluated using repeated 10-fold cross-validation (10 folds, 5 repeats) via the caret package. Variable selection stability and coefficient consistency were evaluated across resampling iterations. The final risk score for each patient was calculated as a linear combination of the selected features weighted by their respective LASSO coefficients. Model calibration was assessed via a calibration curve, and the 95% confidence intervals (CI) for the AUC were calculated using DeLong's method via the pROC package.

**Table S1 Comparison of clinical characteristics between responders and non-responders.**

| **Variable** | **P_Value** | **Non-Response** | **Response** |
| --- | --- | --- | --- |
| Age | 0.825 | Mean: 64.90; Med: 67.00 (IQR: 60.50-72.50) | Mean: 64.90; Med: 67.00 (IQR: 60.50-72.50) |
| BMI | 0.859 | Mean: 22.24; Med: 21.91 (IQR: 20.24-24.30) | Mean: 22.24; Med: 21.91 (IQR: 20.24-24.30) |
| Sex | 0.691 | Female: 4 (30.8%) Male: 9 (69.2%) | Female: 8 (44.4%) Male: 10 (55.6%) |
| ECOG | 0.318 | 0: 5 (38.5%) 1: 6 (46.2%) 2: 2 (15.4%) | 0: 8 (44.4%) 1: 10 (55.6%) |
| Smoke.His | 0.439 | No: 6 (46.2%) Yes: 7 (53.8%) | No: 12 (66.7%) Yes: 6 (33.3%) |
| Alcohol.His | 0.449 | No: 7 (53.8%) Yes: 6 (46.2%) | No: 13 (72.2%) Yes: 5 (27.8%) |
| Family.His | 0.625 | No: 10 (76.9%) Yes: 3 (23.1%) | No: 16 (88.9%) Yes: 2 (11.1%) |
| Position | 0.064 | Gastroesophageal: 3 (23.1%) Only.Stomach: 10 (76.9%) | Only.Stomach: 18 (100.0%) |
| Pathological | 1.000 | Moderately: 2 (15.4%) N.A: 4 (30.8%) Poorly: 6 (46.2%) Poorly.to.Moderately: 1 (7.7%) | Moderately: 2 (11.1%) N.A: 6 (33.3%) Poorly: 8 (44.4%) Poorly.to.Moderately: 2 (11.1%) |
| Lauren | 0.821 | Diffuse: 1 (7.7%) Intestinal: 2 (15.4%) N.A: 10 (76.9%) | Diffuse: 1 (5.6%) Intestinal: 2 (11.1%) Mixed: 2 (11.1%) N.A: 13 (72.2%) |
| Mis.Status | 0.727 | MSS: 7 (53.8%) N.A: 6 (46.2%) | MSS: 12 (66.7%) N.A: 6 (33.3%) |
| Liver.M | 0.233 | No: 5 (38.5%) Yes: 8 (61.5%) | No: 12 (66.7%) Yes: 6 (33.3%) |
| Lymph.node.M | 1.000 | No: 5 (38.5%) Yes: 8 (61.5%) | No: 7 (38.9%) Yes: 11 (61.1%) |
| Peritoneal.M | 1.000 | No: 10 (76.9%) Yes: 3 (23.1%) | No: 14 (77.8%) Yes: 4 (22.2%) |
| Other.M | 0.099 | No: 8 (61.5%) Yes: 5 (38.5%) | No: 16 (88.9%) Yes: 2 (11.1%) |
| M.Site.Num | 0.050 | 1: 5 (38.5%) 2: 5 (38.5%) 3: 3 (23.1%) | 1: 13 (72.2%) 2: 5 (27.8%) |
| Immunotherapy | 0.369 | Yes: 13 (100.0%) | Yes: 18 (100.0%) |
| Chemotherapy | 0.625 | SOX: 10 (76.9%) XELOX: 3 (23.1%) | SOX: 16 (88.9%) XELOX: 2 (11.1%) |
| PD.L1 | 1.000 | Negative: 12 (92.3%) Positive: 1 (7.7%) | Negative: 16 (88.9%) Positive: 2 (11.1%) |

**Table S2 The correlation between IL-15, MUC-16, MMP12 and baseline clinical pathological characteristics.**

| **Variable** | **Group** | **Stats** | **Assoc_P_IL15** | **Assoc_P_MUC.16** | **Assoc_P_MMP12** |
| --- | --- | --- | --- | --- | --- |
| Sex | Female | 12 (38.7%) | 0.617 | 0.889 | 0.952 |
| Sex | Male | 19 (61.3%) |  |  |  |
| Age | Median (IQR) | 67.00 (60.50 - 72.50) | 0.233 | 0.660 | 0.056 |
| BMI | Median (IQR) | 21.91 (20.24 - 24.30) | 0.968 | 0.361 | 0.921 |
| ECOG | 0 | 13 (41.9%) | 0.531 | 0.799 | 0.660 |
| ECOG | 1 | 18 (58.1%) |  |  |  |
| PD.L1 | Negative | 28 (90.3%) | 0.159 | 0.777 | 0.204 |
| PD.L1 | Positive | 3 (9.7%) |  |  |  |
| Smoke.His | No | 18 (58.1%) | 0.708 | 0.567 | 0.622 |
| Smoke.His | Yes | 13 (41.9%) |  |  |  |
| Alcohol.His | No | 20 (64.5%) | 0.792 | 0.338 | 0.157 |
| Alcohol.His | Yes | 11 (35.5%) |  |  |  |
| Family.His | No | 26 (83.9%) | 0.620 | 0.214 | 0.159 |
| Family.His | Yes | 5 (16.1%) |  |  |  |
| Liver.M | No | 17 (54.8%) | 0.356 | 0.544 | 0.109 |
| Liver.M | Yes | 14 (45.2%) |  |  |  |
| Lymph.node.M | No | 12 (38.7%) | 0.484 | 0.164 | 0.826 |
| Lymph.node.M | Yes | 19 (61.3%) |  |  |  |
| Peritoneal.M | No | 24 (77.4%) | 0.661 | 0.115 | 0.764 |
| Peritoneal.M | Yes | 7 (22.6%) |  |  |  |

**Table S3 The Olink Immuno-Oncology panel using in this study.**

| **Protein** | **Uniprot ID** |
| --- | --- |
| Adenosine Deaminase (ADA) | P00813 |
| Adhesion G-protein coupled receptor G1 (ADGRG1) | Q9Y653 |
| Angiopoietin-1 (ANGPT1) | Q15389 |
| Angiopoietin-1 receptor (TIE2) | Q02763 |
| Angiopoietin-2 (ANGPT2) | O15123 |
| Arginase-1 (ARG1) | P05089 |
| Carbonic anhydrase IX (CAIX) | Q16790 |
| Caspase-8 (CASP-8 ) | Q14790 |
| C-C motif chemokine 17 (CCL17) | Q92583 |
| C-C motif chemokine 19 (CCL19) | Q99731 |
| C-C motif chemokine 20 (CCL20) | P78556 |
| C-C motif chemokine 23 (CCL23) | P55773 |
| C-C motif chemokine 3 (CCL3) | P10147 |
| C-C motif chemokine 4 (CCL4 ) | P13236 |
| CD27 antigen (CD27) | P26842 |
| CD40 ligand (CD40-L) | P29965 |
| CD40L receptor (CD40) | P25942 |
| CD70 antigen (CD70) | P32970 |
| CD83 antigen (CD83) | Q01151 |
| C-X-C motif chemokine 1 (CXCL1) | P09341 |
| C-X-C motif chemokine 10 (CXCL10 ) | P02778 |
| C-X-C motif chemokine 11 (CXCL11) | O14625 |
| C-X-C motif chemokine 13 (CXCL13 ) | O43927 |
| C-X-C motif chemokine 5 (CXCL5 ) | P42830 |
| C-X-C motif chemokine 9 (CXCL9 ) | Q07325 |
| Cytotoxic and regulatory T-cell molecule (CRTAM) | O95727 |
| Decorin (DCN) | P07585 |
| Fas antigen ligand (FasL) | P48023 |
| Fibroblast growth factor 2 (FGF2) | P09038 |
| Fractalkine (CX3CL1 ) | P78423 |
| Galectin-1 (Gal-1) | P09382 |
| Galectin-9 (Gal-9) | O00182 |
| Granzyme A (GZMA) | P12544 |
| Granzyme B (GZMB) | P10144 |
| Granzyme H (GZMH) | P20718 |
| Heme oxygenase 1 (HO-1) | P09601 |
| Hepatocyte growth factor (HGF) | P14210 |
| ICOS ligand (ICOSLG) | O75144 |
| Interferon gamma (IFN-gamma) | P01579 |
| Interleukin-1 alpha (IL-1 alpha) | P01583 |
| Interleukin-10 (IL10) | P22301 |
| Interleukin-12 (IL-12) | P29460,P29459 |
| Interleukin-12 receptor subunit beta-1 (IL12RB1) | P42701 |
| Interleukin-13 (IL-13) | P35225 |
| Interleukin-15 (IL15) | P40933 |
| Interleukin-18 (IL-18) | Q14116 |
| Interleukin-2 (IL-2) | P60568 |
| Interleukin-33 (IL-33) | O95760 |
| Interleukin-4 (IL-4) | P05112 |
| Interleukin-5 (IL5) | P05113 |
| Interleukin-6 (IL6) | P05231 |
| Interleukin-7 (IL-7) | P13232 |
| Interleukin-8 (IL-8) | P10145 |
| Killer cell immunoglobulin-like receptor 3DL1 (KIR3DL1) | P43629 |
| Latency-associated peptide transforming growth factor beta-1 (LAP TGF-beta-1) | P01137 |
| Lymphocyte activation gene 3 protein (LAG3) | P18627 |
| Lysosome-associated membrane glycoprotein 3 (LAMP3) | Q9UQV4 |
| Macrophage colony-stimulating factor 1 (CSF-1) | P09603 |
| Matrix metalloproteinase-12 (MMP-12) | P39900 |
| Matrix metalloproteinase-7 (MMP-7) | P09237 |
| MHC class I polypeptide-related sequence A/B (MIC-A/B) | Q29983,Q29980 |
| Monocyte chemotactic protein 1 (MCP-1) | P13500 |
| Monocyte chemotactic protein 2 (MCP-2) | P80075 |
| Monocyte chemotactic protein 3 (MCP-3) | P80098 |
| Monocyte chemotactic protein 4 (MCP-4) | Q99616 |
| Mucin-16 (MUC-16) | Q8WXI7 |
| Natural cytotoxicity triggering receptor 1 (NCR1) | O76036 |
| Natural killer cell receptor 2B4 (CD244) | Q9BZW8 |
| Natural killer cells antigen CD94 (KLRD1) | Q13241 |
| Nitric oxide synthase, endothelial (NOS3) | P29474 |
| Placenta growth factor (PGF) | P49763 |
| Platelet-derived growth factor subunit B (PDGF subunit B) | P01127 |
| Pleiotrophin (PTN) | P21246 |
| Pro-epidermal growth factor (EGF) | P01133 |
| Programmed cell death 1 ligand 1 (PD-L1) | Q9NZQ7 |
| Programmed cell death 1 ligand 2 (PD-L2) | Q9BQ51 |
| Programmed cell death protein 1 (PDCD1) | Q15116 |
| Stromal cell-derived factor 1 (CXCL12) | P48061 |
| T-cell surface glycoprotein CD4 (CD4) | P01730 |
| T-cell surface glycoprotein CD5 (CD5) | P06127 |
| T-cell surface glycoprotein CD8 alpha chain (CD8A) | P01732 |
| T-cell-specific surface glycoprotein CD28 (CD28) | P10747 |
| TNF-related apoptosis-inducing ligand (TRAIL) | P50591 |
| Tumor necrosis factor (Ligand) superfamily, member 12 (TWEAK) | O43508 |
| Tumor necrosis factor (TNF) | P01375 |
| Tumor necrosis factor ligand superfamily member 14 (TNFSF14 ) | O43557 |
| Tumor necrosis factor receptor superfamily member 12A (TNFRSF12A) | Q9NP84 |
| Tumor necrosis factor receptor superfamily member 21 (TNFRSF21) | O75509 |
| Tumor necrosis factor receptor superfamily member 4 (TNFRSF4 ) | P43489 |
| Tumor necrosis factor receptor superfamily member 9 (TNFRSF9) | Q07011 |
| Vascular endothelial growth factor A (VEGF-A) | P15692 |
| Vascular endothelial growth factor receptor 2 (VEGFR-2) | P35968 |

**Fig. S1: Distribution of baseline NPX values of IL-15, MUC-16, and MMP12, and baseline IL-15 stratified by clinical variables.**

(a–c) The distribution of baseline NPX values for IL-15, MUC-16, and MMP12 in the study cohort. (d) Baseline IL-15 NPX levels stratified according to clinical characteristics, including sex, alcohol consumption history (Alcohol.His), smoking history (Smoke.His), family history of cancer (Family.His), lymph node metastasis (Lymph.node.M), peritoneal metastasis (Peritoneal.M), programmed death-ligand 1 (PD-L1) status. Associations between baseline IL-15 levels and continuous clinical variables, including age and body mass index (BMI).

**
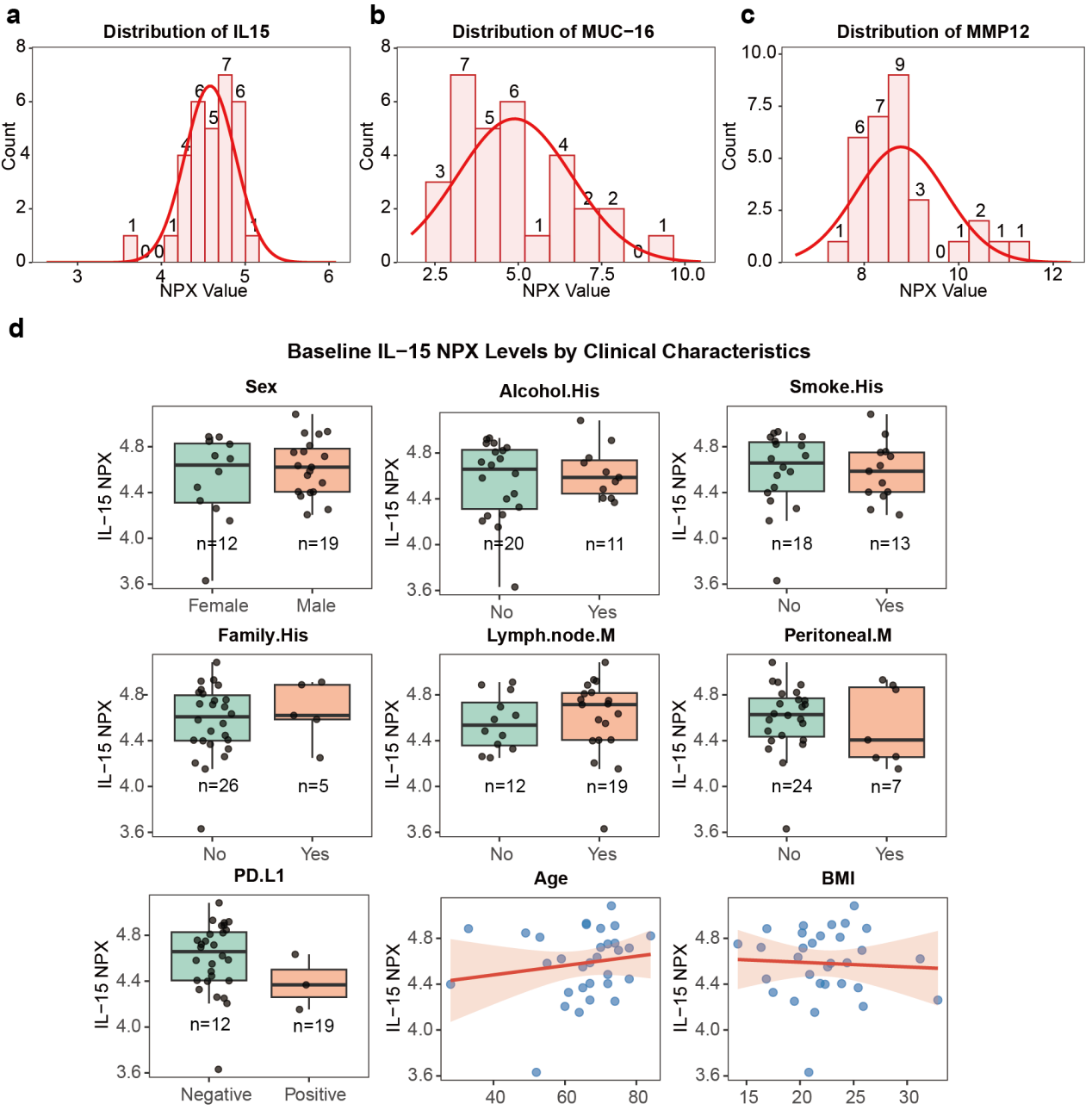
**

**Fig. S2: Peripheral proteins expression correlate with prognostic survival at T0, T1, T2, T0-T1, and T1-T2.**

(a) Baseline (T0) protein expression levels associated with survival outcomes. Adhesion G protein-coupled receptor G1 (ADGRG1), colony stimulating factor 1 (CSF1), interleukin-4 (IL-4), interleukin-6 (IL-6), and interleukin-33 (IL-33). (b) Protein expression levels at T1 associated with survival outcomes. Colony-stimulating factor 1 (CSF1), C-X-C motif chemokine ligand 9 (CXCL9), interleukin-1 alpha (IL-1α), interleukin-15 (IL-15), latency-associated peptide transforming growth factor beta 1 (LAP TGFβ1), interleukin-2 (IL-2), interleukin-5 (IL-5), interleukin-10 (IL-10), monocyte chemoattractant protein-3 (MCP3), TNF superfamily member 14 (TNFSF14). (c) Protein expression levels at T2 associated with survival outcomes. C-C motif chemokine ligand 3 (CCL3), cluster of differentiation 4 (CD4), galectin-9 (Gal9), heme oxygenase 1 (HO1), monocyte growth factor (MGF), TNF receptor superfamily member 9 (TNFRSF9), TNF superfamily member 14 (TNFSF14). (d) Dynamic changes in protein expression between T0 and T1 associated with survival outcomes. Interleukin-4 (IL-4), interleukin-10 (IL-10), monocyte chemoattractant protein-2 (MCP2). (e) Dynamic changes in protein expression between T1 and T2 associated with survival outcomes. Granzyme B (GZMB), monocyte chemoattractant protein-3 (MCP3).

**
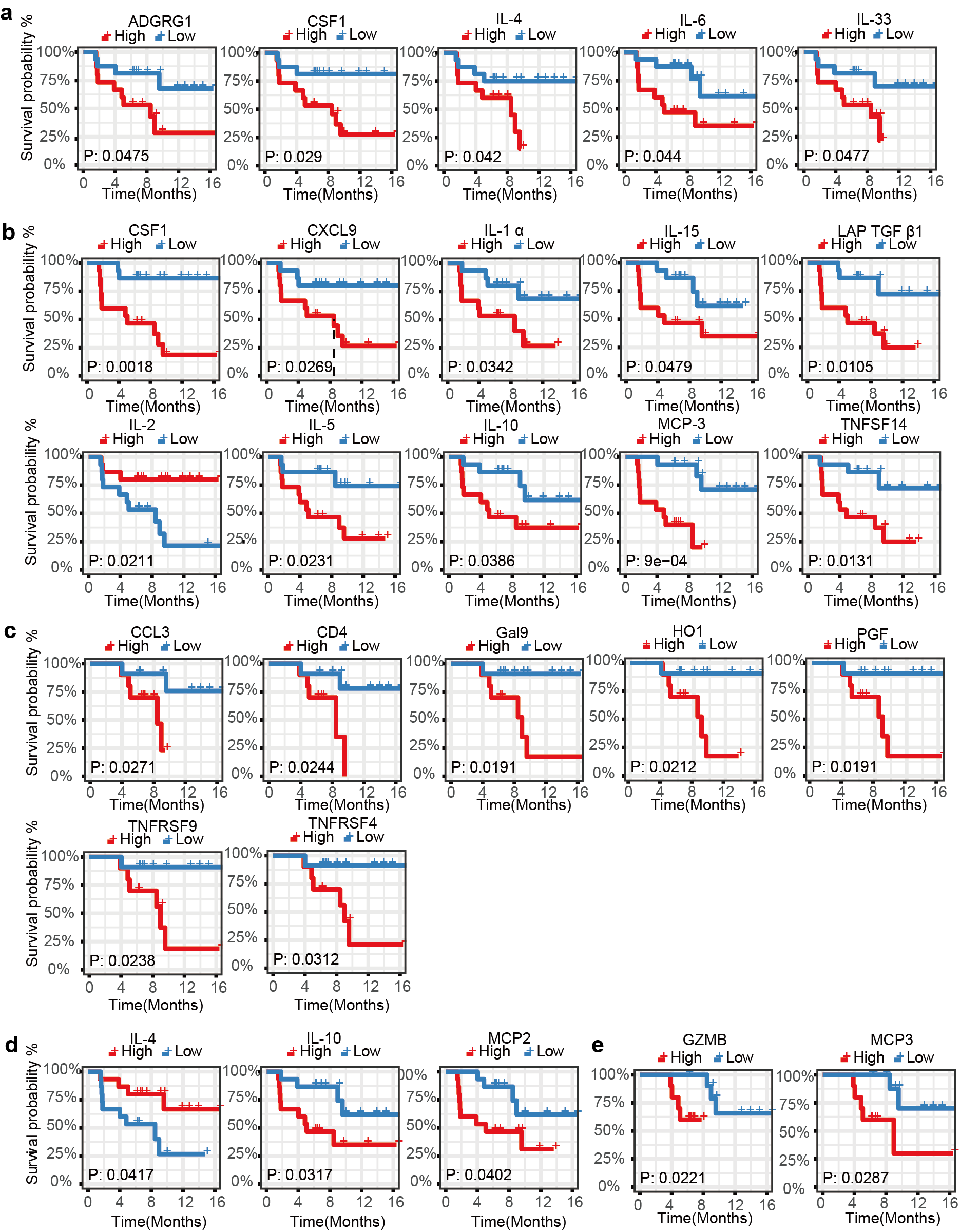
**

**Fig. S3: Pairwise correlations between target plasma proteins and clinical inflammatory/infection markers at T0 and T1.**

(a) Correlation analysis between target plasma proteins (MUC-16, MMP12, IL-15) and clinical inflammatory/infection-related markers (CA125, NE, PCT, WBC) at baseline (T0). (b) Correlation analysis between target plasma proteins (MUC-16, MMP12, IL-15) and clinical inflammatory/infection-related markers (CA125, NE, PCT, WBC) at T1. Mucin-16 (MUC-16), matrix metalloproteinase-12 (MMP12), interleukin-15 (IL-15), cancer antigen 125 (CA125), neutrophil ratio (NE), procalcitonin (PCT), white blood cell count (WBC). * *P* < 0.05.

**
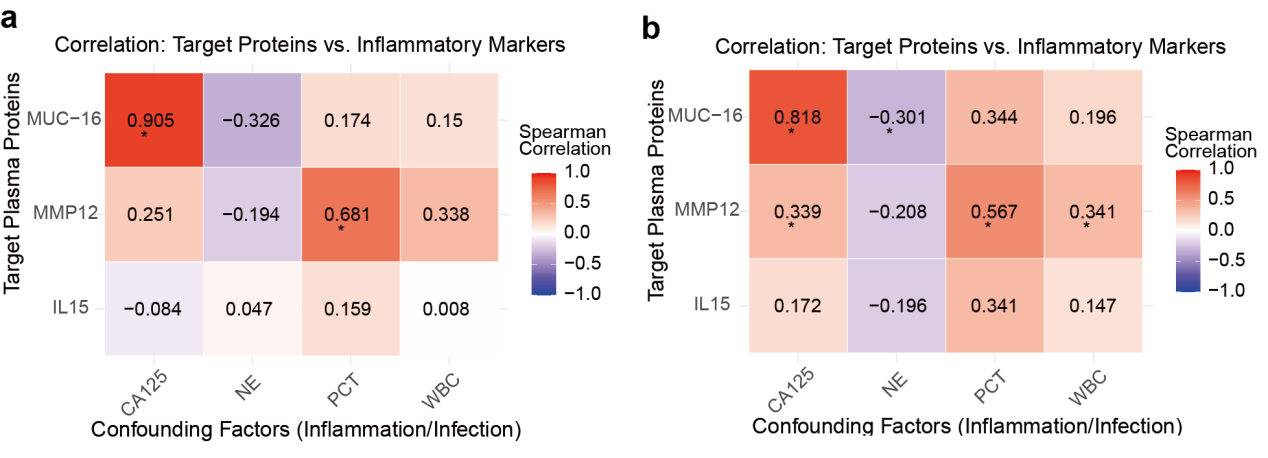
**

**Fig. S4: GO enrichment heatmap and Drug-gene interaction network.**

(a) Significantly enriched biological process (GO-BP), cellular component (GO-CC), and molecular function (GO-MF) terms associated with the identified proteins. (b) Drug–gene interaction network constructed based on the identified proteins and their associated therapeutic agents. Pink nodes, genes; blue nodes, drugs.**
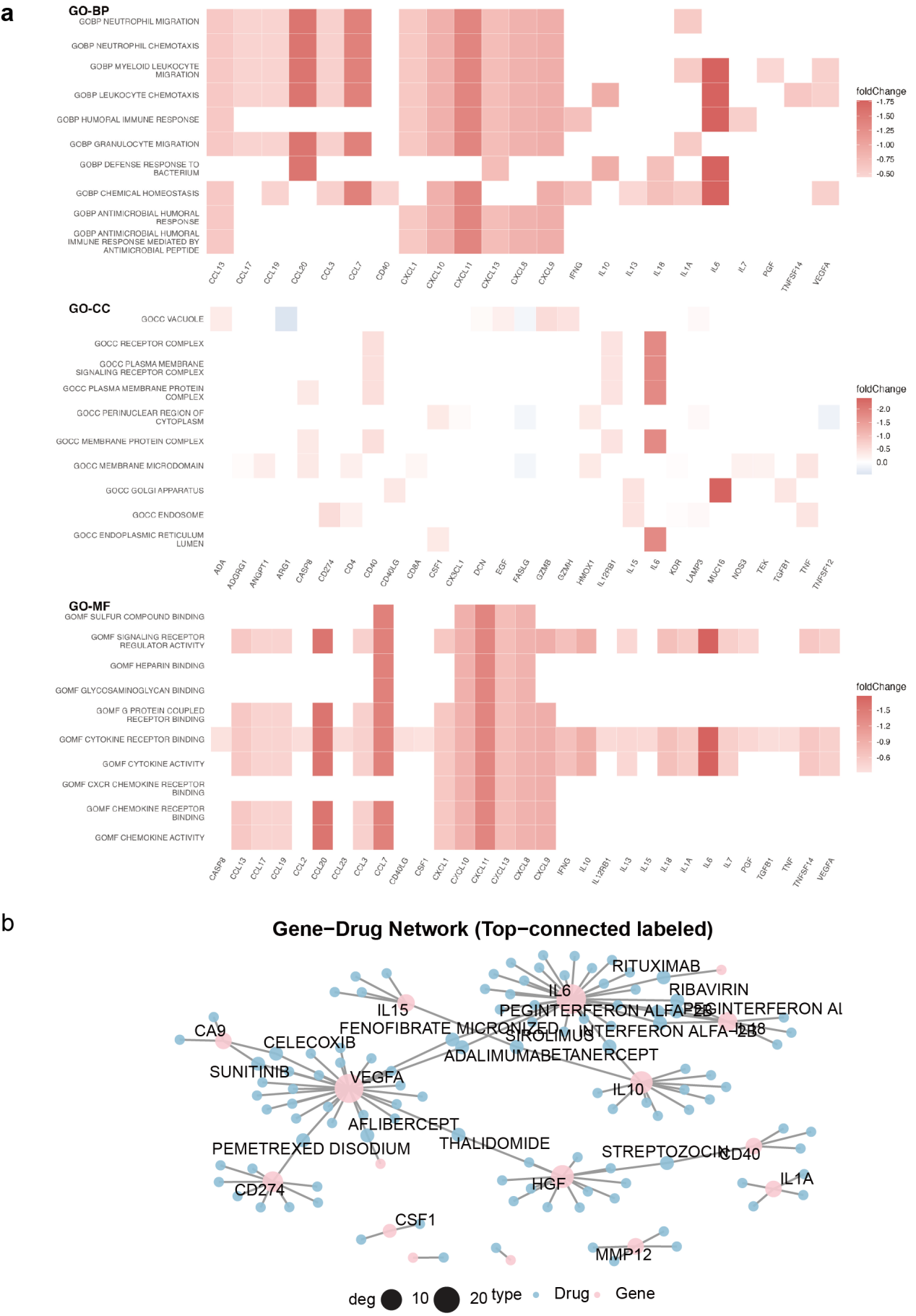
**
